# Pure yolk sac tumor of the testis on histopathology: a clinical image

**DOI:** 10.11604/pamj.2024.49.8.44804

**Published:** 2024-09-05

**Authors:** Shivali Kalode, Prerna Tekulwar

**Affiliations:** 1 Department of Pathology, Jawaharlal Nehru Medical College, Datta Meghe Institute of Higher Education and Research, Sawangi (Meghe), Wardha, Maharashtra, India

**Keywords:** Yolk sac tumor, testis, Schiller-Duval bodies, clinical image

## Image in medicine

Testicular cancer makes up only 1% of all male cancers. Pure yolk sac tumor of the testis accounts for 0.6% of testicular germ cell tumors. Risk factors include contralateral tumor occurrence, infertility, exposure to diethylstilbestrol, and family history of testicular tumors in first-degree and undescended testis. Here we are presenting a case of a 30-year-old male, who presented with a chief complaint of a gradually progressive, mildly tender, gradually growing abdominal lump which was insidious in onset for the last one month. Associated with a few episodes of non-projectile, non-bilious vomiting and delayed bowel movement for the last 10 days. Personal history includes unilateral cryptorchidism and infertility. On examination, single, ovoid, non-tender, firm, non-ballottable, abdominal lump of size approximately 15 x 12 x 5 cm was identified. Percussion revealed a tympanic note in the supra-umbilical region and a dull note in the infra-umbilical region. Examination of the scrotum revealed the absence of one testis. Further, an ultrasound-guided tru-cut biopsy favored the diagnosis of a pure yolk sac tumor of the testis. The patient underwent exploratory laparotomy and the testis along with the spermatic cord was removed and sent to the histopathology section. Excised specimen of orchidectomy with an attached spermatic cord measuring 18 x 15 x 5 cm. On the cut section, a greyish-brown, circumscribed, unencapsulated solid mass was identified. Microscopically, H&E staining revealed Schiller-Duval bodies, hyaline globules, areas of necrosis, and hemorrhage were noted, features suggestive of a pure yolk sac tumor of the testis. Post-operative recovery was uneventful and further advised to keep follow-up every month.

**Figure 1:**
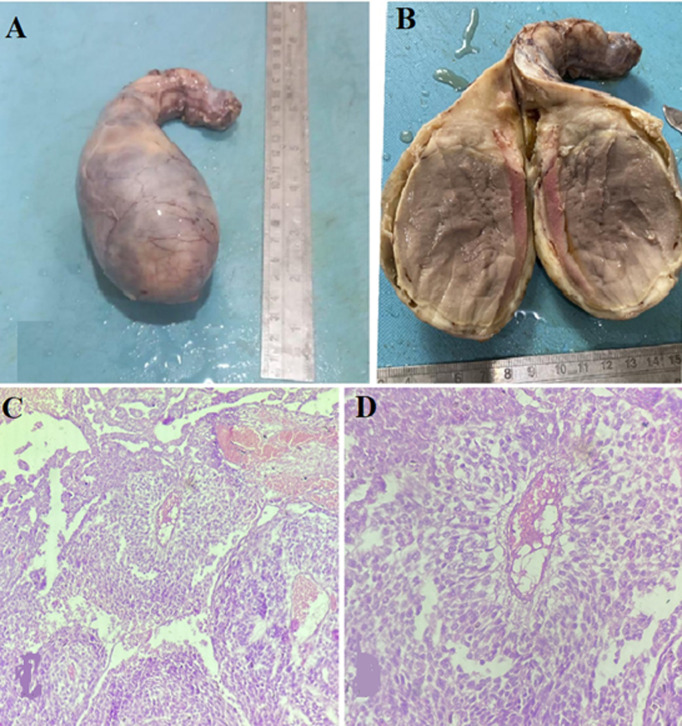
A) orchidectomy with an attached spermatic cord; B) cut surface showing a greyish-brown, circumscribed, unencapsulated solid mass; (C,D) microscopic image at low power (10X) and high power view (40X) showing H&E staining revealing Schiller-Duval bodies, hyaline globules, areas of necrosis, and hemorrhage

